# Gut microbiota–cholesterol crosstalk in cardiovascular diseases: mechanisms, metabolites, and therapeutic modulation

**DOI:** 10.1186/s12986-025-01051-7

**Published:** 2025-12-04

**Authors:** Mohammad Abavisani, Seyed Mohammad Sajjadi, Negar Ebadpour, Sercan Karav, Amirhossein Sahebkar

**Affiliations:** 1 Student research committee, Mashhad University of Medical Sciences, Mashhad, Iran; 2 Immunology Research Center, Mashhad University of Medical Sciences, Mashhad, Iran; 3 Department of Molecular Biology and Genetics, Canakkale Onsekiz Mart University, Canakkale, 17100 Turkey; 4 Biotechnology Research Center, Pharmaceutical Technology Institute, Mashhad University of Medical Sciences, Mashhad, Iran; 5 Centre for Research Impact & Outcome, Chitkara College of Pharmacy, Chitkara University, Rajpura, 140401 Punjab India; 6 Applied Biomedical Research Center, Basic Sciences Research Institute, Mashhad University of Medical Sciences, Mashhad, Iran

**Keywords:** Atherosclerosis, Cholesterol, Gastrointestinal microbiome, Homeostasis

## Abstract

Cardiovascular diseases (CVD) are one of the leading causes of death worldwide. Genetic factors, and various environmental factors, including nutrition and the composition of the gut microbiota, have been identified as important factors in the initiation of CVD. Among them, the pivotal role of the gut microbiota in modulating cholesterol metabolism and influencing cardiovascular outcomes has recently been highlighted. Extensive research has confirmed that the gut microbiota has direct and indirect regulatory effects on host cholesterol homeostasis. Recent studies have shown that the microbiota can influence blood cholesterol levels and thus the risk of CVD through various pathways, such as the production of certain metabolites such as bile acids (BAs), SCFAs, and TMAO, the activation of nuclear and membrane-bound receptors such as farnesoid X receptor (FXR), the regulation of gene expression involved in lipid metabolism and inflammatory responses, as well as microbial enzymatic pathways. These complex regulatory mechanisms make the gut microbiota a potential therapeutic target in cholesterol-related diseases and CVD. Microbiota-modulating strategies, including the use of probiotics, prebiotics, fecal microbiota transplantation (FMT), and selective antibiotics, have shown beneficial effects in previous studies. In this regard, in this study, we conducted an in-depth investigation of the regulatory effect of intestinal microbiota on cholesterol metabolism and their impact on the development and progression of atherosclerosis and CVD, and described potential therapeutic pathways based on the regulation of intestinal microbiota in CVD.

## Introduction

 Cardiovascular diseases (CVD), particularly those driven by atherosclerosis (AS), remain a leading cause of global morbidity and mortality [1]. Beyond genetic predispositions, environmental factors, specifically nutrition and the composition of the intestinal microbiota, have been identified as critical contributors to the onset of CVD [2]. The gut microbiome, consisting of a highly diverse and complex population of trillions of microorganisms, serves as a fundamental regulator of host metabolic activity. It significantly contributes to various physiological processes, including the host’s metabolic regulation, immune system modulation, carbohydrate digestion, and the generation of bioactive signaling compounds such as short-chain fatty acids (SCFAs) and bile acids (BAs) [3, 4]. The maintenance of microbial equilibrium within the gut is essential for sustaining systemic homeostasis. Conversely, disruption of this microbial balance (dysbiosis) can provoke immune dysregulation, thereby playing a pivotal role in the onset and progression of CVD, including AS [5].

Hypercholesterolemia, a well-established risk factor, has long been known as an integral risk factor implicated in the pathogenesis of atherosclerotic CVD. Although several classes of lipid-lowering agents have been introduced for the management of hypercholesterolemia hypercholesterolemia [6–9], the burden of the problem is still high [6]. Statins, which are the cornerstone of hypercholesterolemia treatment, have established efficacy in reducing cardiovascular events [10] and possess myriad pleiotropic actions [11–16], yet a considerable proportion of individuals cannot tolerate these drugs or reach therapeutic low-density lipoprotein-cholesterol (LDL-C) targets despite receiving treatment [17]. This situation poses a real need for additional tools to be used as either adjuncts or alternatives to statins.

Recent studies have highlighted that one of the primary pathways for cholesterol elimination involves its conversion to BAs and subsequent excretion. Commensal gut microbiota facilitates the transformation of primary BAs into secondary BAs, such as deoxycholic acid (DCA), ursocholic acid, ursodeoxycholic acid (UDCA), and lithocholic acid (LCA) [18, 33, 34]. Accumulating evidence suggests a strong association between BA metabolism and the development and progression of AS [19]. Conversely, dysbiosis-induced alterations in BA composition can impair both farnesoid X receptor (FXR) and Takeda G protein-coupled receptor 5 (TGR5) signaling, thereby aggravating lipid metabolism disorders and vascular inflammation, which are central to the pathophysiology of AS and CVD [20, 21]. Moreover, the fermentation of dietary fibers by gut microbiota produces SCFAs. Indeed, the microbial metabolite trimethylamine N-oxide (TMAO), which originates from the microbial metabolism of dietary choline and carnitine, converts into trimethylamine (TMA), which is subsequently oxidized in the liver to TMAO. Elevated plasma TMAO levels have been associated with increased cholesterol accumulation in arterial walls, foam cell formation, and platelet hyperactivity—hallmark processes in the development of AS [22].

The identification of gut microbiota-derived metabolic signatures holds potential for the development of predictive biomarkers for cardiovascular events. Promising microbiota-modulating strategies, including the use of probiotics, prebiotics, and fecal microbiota transplantation (FMT), have shown beneficial effects [23, 24]. In this regard, we provided an in-depth review on the regulatory impact of gut microbiota on cholesterol metabolism and their effect on the development and progression of AS and CVD, aimed at offering novel therapeutic targets for intervention.

## Gut microbiota and cholesterol metabolism

### BA metabolism

**Fig. 1:**
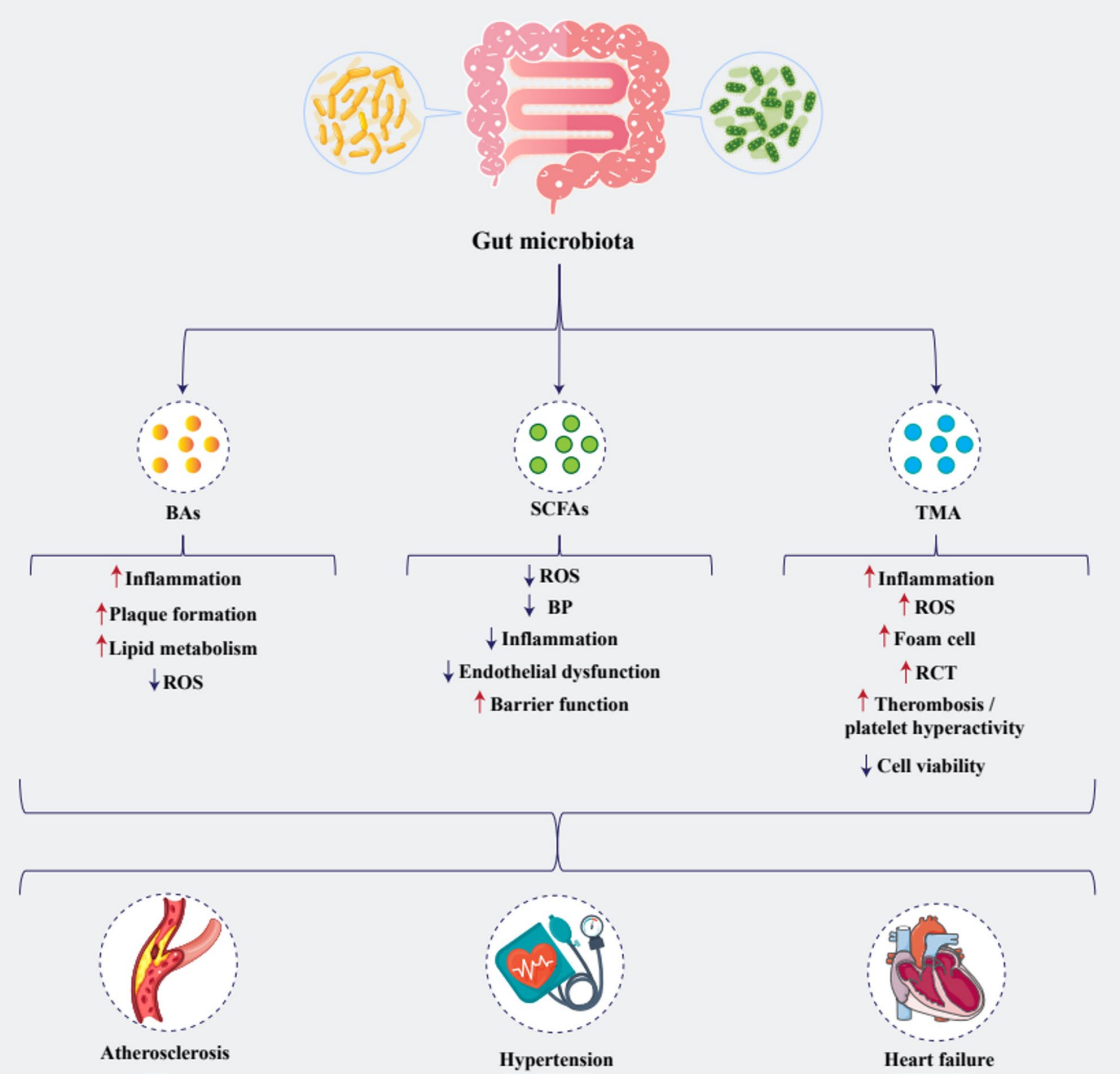
Differential effects of gut microbial metabolites on cardiovascular health. Gut microbial metabolites—including bile acids (BAs), short-chain fatty acids (SCFAs), and trimethylamine (TMA)—exert divergent effects on vascular and cardiac function. BAs drive pro-atherogenic processes by increasing inflammation, plaque formation, and lipid metabolism while reducing reactive oxygen species (ROS), thereby promoting atherosclerosis. In contrast, SCFAs confer cardioprotection through lowering ROS, blood pressure (BP), inflammation, and endothelial dysfunction and by enhancing gut barrier integrity, mitigating hypertension. Conversely, TMA amplifies inflammation, oxidative stress, foam cell formation, reverse cholesterol transport, and thrombosis while impairing cell viability, contributing to heart failure

One of the major pathways for cholesterol elimination from the body is via BA excretion. Beyond their synthesis and enterohepatic circulation, BAs regulate triglyceride, cholesterol, glucose metabolism, and overall energy homeostasis by activating multiple signaling pathways (Fig. 1) [18]. Additionally, they are essential for the absorption of lipid-soluble vitamins and dietary lipids [25]. Increasing evidence supports a close association between BA metabolism and the onset and progression of AS [19]. BAs are synthesized from cholesterol via two pathways: the classical (or neutral) pathway, which occurs in hepatocytes, and the alternative (or acidic) pathway, which is initiated in extrahepatic tissues and completed in the liver [26]. The conventional pathway is regarded as the predominant route of BA synthesis since it facilitates the synthesis of primary BA via the rate-limiting cholesterol 7α-hydroxylase (CYP7A1) enzyme and is responsible for over 90% of BA synthesis [27]. This process is initiated by the rate-limiting enzyme CYP7A1, which catalyzes the 7α-hydroxylation of cholesterol [28]. On the other hand, less than 10% of BA synthesis is mediated by the alternative pathway [29].

Primary BAs synthesized in the liver are conjugated with taurine or glycine and stored in the gallbladder [30, 31]. Upon meal consumption, they are secreted into the duodenum via the biliary system to facilitate lipid emulsification. While most BAs are reabsorbed in the ileum and returned to the liver via the enterohepatic circulation, a small proportion escapes reabsorption and reaches the colon. Reabsorption of BAs in the distal intestine contributes to the formation of a concentrated reservoir of these molecules, referred to as the BA pool [32]. There, gut commensal microorganisms convert primary BAs into secondary BAs such as deoxycholic acid (DCA), ursocholic acid, ursodeoxycholic acid (UDCA), and lithocholic acid (LCA) []. Generally, this transformation Increased variety and a more hydrophobic BA pool are the results of microbial metabolism of BAs that promotes their excretion in feces [35]. To maintain the size of the BA pool under steady-state circumstances, hepatic de novo synthesis compensates for this loss [36]. DCA and LCA are the predominant secondary BAs, generated through microbial 7α-dehydroxylation of cholic acid (CA) and CDCA, respectively. While DCA is largely reabsorbed and reconjugated into the BA pool, LCA is often sulfonated in the liver to facilitate its elimination, which facilitates its removal from the body [37].

### BA signaling via FXR and TGR5

**Fig. 2:**
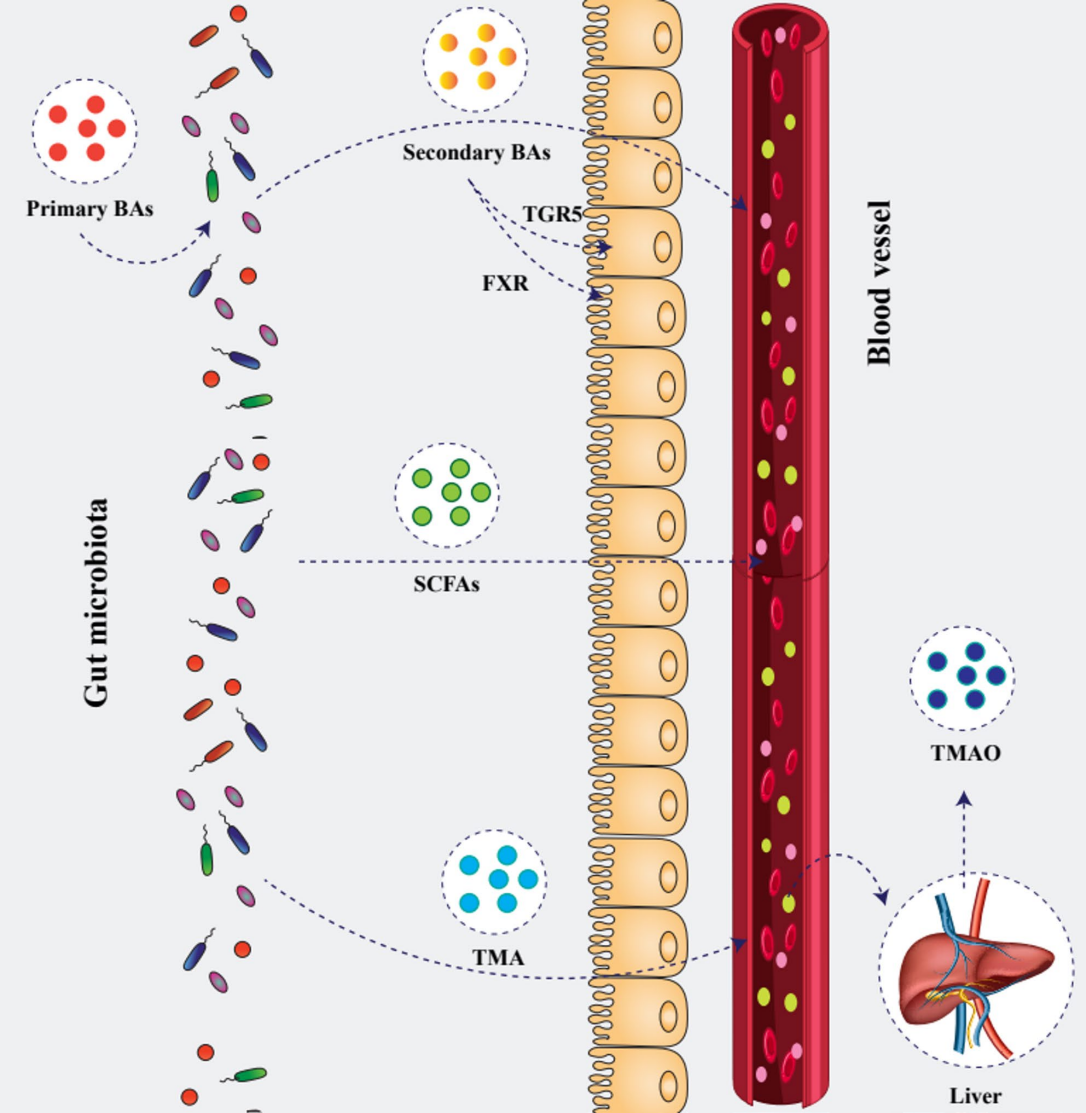
Gut microbial metabolites crossing the intestinal barrier into systemic circulation. Gut bacteria convert host-derived primary BAs into secondary BAs, which engage the nuclear receptor FXR and the G‐protein–coupled receptor TGR5 on enterocytes before entering the bloodstream. SCFAs, produced by microbial fermentation of dietary fibers, diffuse across the epithelium to exert systemic effects on metabolism and inflammation. Microbial TMA is absorbed into portal blood and oxidized by the liver into trimethylamine N-oxide (TMAO). Once in the circulation, these microbial metabolites act on distant organs, including the cardiovascular system, to influence host physiology

It has recently been shown that BAs are signaling molecules for a range of functions that are mediated through the TGR5 and the FXR (Fig. 2) [38]. These receptors coordinate signaling pathways that impact metabolic, inflammatory, and immune responses. FXR, a nuclear receptor predominantly found in the liver and intestines, is stimulated by particular BA and acts as a key regulator of BA production, liver triglyceride lipid metabolism, and glucose regulation [39, 40]. Among BAs, CDCA is the most potent natural FXR agonist, while CA exhibits the weakest activity [41]. BAs have the ability to activate the nuclear receptor FXR once they are within enterocytes. FXR then dimerizes with the retinoid X receptor (RXR) inside the nucleus to connect with DNA at the FXR response elements (FXRRE) and change gene transcription [42]. As a result, these interactions cause the transcription of many genes involved in the transcellular transport of BAs and human fibroblast growth factor (FGF) 19 to be activated [43]. FGF19 is released into the portal circulation and subsequently reaches the liver, where it binds to and activates the FGF receptor 4/β-Klotho (FGFR4/KLB) complex. This activation initiates signaling via the docking protein fibroblast growth factor receptor substrate 2 α (FRS2α) and the Src homology-2 domain-containing protein tyrosine phosphatase-2 (Shp2), resulting in the phosphorylation of extracellular signal-regulated kinases (ERK) 1/2. The expression of the CYP7A1 gene, controlled by the nuclear factors hepatocyte nuclear factor 4α (HNF4α) and liver receptor homolog-1 (LRH-1), is suppressed, leading to a decrease in BA production [44, 45].

TGR5 has strong expression in several organs, including the liver, lung, and small intestine, and is also found in the heart [46]. TGR5 has the same comparable rankings for potency and effectiveness, which are LCA >DCA >CCA >CA [20]. Maintaining energy homeostasis, having immunosuppressive qualities, and controlling glucose and lipid metabolism are the key TGR5-dependent functions of BAs [47]. TGR5-regulated macrophage polarization has a direct impact on the propensity for an inflammatory response [48]. By increasing the ubiquitination of the NLRP3 inflammasome, BA-induced TGR5 activation decreased inflammation [49]. TGR5 signaling impacts various pathways, including AKT, ERK, and NF-κB [50]. Furthermore, TGR5’s immunomodulatory effects have been suggested to contribute to the prevention of AS [51].

### Microbial modifications of BAs

Gut microbiota significantly influences BA composition through various enzymatic modifications, including deconjugation, dehydroxylation, and epimerization, which increases BA variety and impacts host physiology.

#### Deconjugation

In the deconjugation process of secondary BAs, a unique enzyme called bile salt hydrolase (BSH), which is often present in bacteria that live in the colon and small intestine, hydrolyzes the amide bond of the conjugated BAs, transforming them into their unconjugated counterparts, thereby influencing the enterohepatic circulation and increasing the BA pool’s variety [35, 52]. BSH activity is present in all major bacterial phyla of the gut, with key contributors including *Bacteroides fragilis*, *Bacteroides vulgatus*, *Clostridium perfringens*, *Listeria monocytogenes*, *Lactobacillus*, and *Bifidobacterium* species [53, 54].

#### Dehydroxylation

The ratio of secondary BAs to primary BAs showed a positive correlation with the *baiE* gene abundance of cluster 1 [55]. The *bai* operon contains genes that encode several proteins involved in catalyzing the 7-dehydroxylation process [56]. It has been reported that in the human intestine, *Clostridium* is able to encode the proteins required for the 7α-dehydroxylation process [57]. In a different study, *C. sordellii* was classified as a “low activity” strain, but species like *C. scindens* exhibited 100 times higher BA 7α-dehydroxylating activity [58].

#### Epimerization

In addition to deconjugation and dehydroxylation, microbial enzymes facilitate the epimerization and oxidation of hydroxyl groups at various positions on the BA nucleus. The hydroxysteroid dehydrogenases (HSDHs) of intestinal bacteria are responsible for the oxidation and epimerization of the 3-, 7-, or 12-hydroxyl groups of BAs [59]. The epimerization of BA hydroxyl groups represents a reversible alteration in stereochemistry, transitioning from the α to the β configuration, accompanied by the formation of a stable carbonyl group BA intermediate. This procedure involves two separate steps: the oxidation of the hydroxyl group by a position-specific HSDH and the subsequent reduction of the hydroxyl group by another position-specific HSDH [60]. HSDH activity has been validated in a wide range of bacteria, such as *Bacteroides*,* Eubacterium*,* and Clostridium* [61]. The reactions can occur in a co-culture of multiple organisms, leading to a rich chemical diversity of secondary BAs [62]. CA may be epimerized to provide derivatives such as UCA, 12-epicholic acid, or isocholic acid, while CDCA can be epimerized to produce either UDCA or isochenodeoxycholic acid [63, 64]. Epimerization diminishes the toxicity of the final pool of BAs by decreasing the molecules with α-oriented hydroxyl groups and limiting the synthesis of dehydroxylated BAs [60]. An appropriate balance between primary and secondary BAs is critical for metabolic health, as excessive accumulation of secondary BAs has been implicated in diseases such as colorectal cancer [65]. Additionally, patients with chronic heart failure (HF) display altered BA profiles, including reduced primary BAs and increased levels of specific secondary BAs [66].

### Microbial production of SCFAs

SCFAs are the major end-products of the microbial fermentation of dietary fibers in the colon. Among them, acetate is the most abundant in systemic circulation, followed by propionate and butyrate [67, 68]. Other microbial metabolites such as lactate isomers, valerate, and branched-chain SCFAs are also present but at considerably lower concentrations [69, 70]. SCFAs participate in certain metabolic activities, including lipid synthesis, fat storage, glucose absorption, and inflammation [71]. They are also necessary for maintaining gut microbiota, controlling immunity, and preserving water and electrolyte balance [72]. Emerging evidence suggests that SCFAs may also influence CVD, including HF (Fig. 1) [73]. Through interactions with G-protein-coupled receptors (GPRs) and modulation of the renin-angiotensin system, SCFAs may regulate blood pressure (BP). Additionally, they improve lipid metabolism, attenuate systemic inflammation, and enhance endothelial function, thereby potentially reducing the risk of AS and hypertension (HTN). SCFAs also strengthen gut barrier integrity, preventing the translocation of pro-inflammatory endotoxins that could exacerbate CVD [74, 75].

SCFAs are predominantly synthesized through microbial fermentation of non-digestible carbohydrates, including dietary fibers and non-starch polysaccharides [76, 77]. The primary bacteria responsible for the production of SCFAs are the clostridial clusters IV and XIVa of Firmicutes, which include species such as *Eubacterium*,* Roseburia*,* Faecalibacterium*, and *Coprococcus* [25]. While butyrate is the principal metabolic byproduct of the *Firmicutes* phylum, the *Bacteroidetes* phylum mostly generates acetate and propionate [70, 78].

#### Effects of SCFAs on cholesterol levels

SCFAs have demonstrated cholesterol-lowering effects in both experimental and clinical studies [79]. Evidence regarding butyrate’s effect on sterol regulatory element-binding protein 2 (SREBP2) signaling is somewhat divergent, likely reflecting model- and context-dependent regulation. In cultured hepatic cells, butyrate acts as a histone deacetylase (HDAC) inhibitor, reducing intracellular cholesterol synthesis by impairing SREBP2 signaling and downregulating SREBP2 target genes, including HMGCR and LDLR [80]. In contrast, in vivo studies in hamsters have shown that dietary butyrate increases hepatic expression of SREBP2, low-density lipoprotein receptor (LDL-R), and CYP7A1, leading to enhanced BA excretion and clearance of circulating cholesterol [81]. Additionally, butyrate impairs intestinal cholesterol absorption by downregulating Niemann–Pick C1-Like 1 (NPC1L1), a key transporter for cholesterol uptake in enterocytes [82]. Together, these findings indicate that butyrate modulates cholesterol metabolism through multiple mechanisms that may differ across experimental systems but converge on lowering circulating cholesterol.

Propionate also exerts hypocholesterolemic effects, primarily by mildly inhibiting HMG-CoA reductase, the rate-limiting enzyme in cholesterol synthesis [83]. It is also able to inhibit the ^14^C incorporation into fatty acid and cholesterol at a modest dosage of 1 mmol oleate/l [84]. Haghikia et al. demonstrated significant effects of propionate treatment in reducing total cholesterol (TC), very-low-density lipoprotein (VLDL), and low-density lipoprotein cholesterol (LDL-C), as well as in preventing AS in hypercholesterolemic ApoE−/− mice. Subsequently, a randomized controlled trial validated the lipid-lowering effect of propionate in humans. After 8 weeks of oral administration of 500 mg BID propionate to individuals with hyperlipidemia, various blood lipid components exhibited notable reductions in TC, LDL-C, and non-HDL cholesterol [85]. Collectively, several studies point towards an important role of SCFAs in cholesterol levels. Experimental findings indicate that SCFAs can influence both cholesterol synthesis and clearance. Notably, the gut microbiota effects in this process are significant, as it is responsible for the fermentation of dietary fibers into SCFAs. Alterations in the gut microbial community can therefore significantly impact host lipid metabolism and cardiovascular risk. However, conflicting data on their precise mechanisms of action remain a significant challenge.

### Atherogenic effects of TMAO

TMAO is a gut microbiota-derived metabolite with established proatherogenic properties. It is synthesized following the microbial metabolism of dietary nutrients rich in choline and carnitine abundant in red meat, eggs, fish, and dairy products into trimethylamine (TMA), which is subsequently oxidized to TMAO [86]. There are four distinct microbial enzymatic pathways that contribute to TMA formation: betaine reductase, TMAO reductase, choline-TMA lyase (CutC/D), and carnitine monooxygenase (CntA/B). Additionally, the YeaW/X enzyme complex, structurally similar to CntA/B, may facilitate TMA production from a broader range of substrates [87]. Notably, the CutC/CutD pathway, driven by gut microbes, significantly contributes to intestinal TMA synthesis in humans [88].

*Anaerococcus hydrogenalis*,* Clostridium asparagiforme*,* Clostridium hathewayi*,* Clostridium sporogenes*,* Edwardsiella tarda*,* Escherichia fergusonii*,* Proteus penneri*, and *Providencia rettger* are among the eight different bacterial strains that have been shown to participate in TMA synthesis [89]. In healthy individuals, the Firmicutes-to-Bacteroidetes ratio within the TMA-producing gut microbiota is approximately 2:1 [90]. Hepatic flavin-containing monooxygenase (FMO)3 and FMO1 oxidized TMA, which was generated specifically in the first section of the colon, after it had been absorbed into the portal circulation [91, 92]. TMAO may then be released by the liver and either absorbed by extrahepatic tissues or eliminated by the kidneys [93]. Some studies have indicated that TMAO may be a marker to predict the risk of CVD, such as ACS and stroke, most likely through a proatherogenic pathway [94–96]. Stable isotope dilution liquid chromatography tandem mass spectrometry has shown a strong correlation between the coronary atherosclerotic load and plasma TMAO levels in individuals with ST-segment elevation myocardial infarction (STEMI) [97]. Similarly, a case–control study showed the correlation between plasma level of TMAO with the risk and severity of coronary heart disease (CHD) [98]. Also, an increased risk of significant adverse cardiac events was linked to increasing TMAO levels, according to research by Tang et al. [99].

Mechanistically, TMAO impairs cholesterol homeostasis by downregulating cholesterol 7α-hydroxylase (CYP7A1) expression by 38.4% in animal models, which suppresses BA synthesis and decreases reverse cholesterol transport (RCT). This process is likely mediated via activation of the FXR and small heterodimer partner (SHP), which inhibit CYP7A1 transcription [100, 101]. Macrophages absorb TMAO molecules by binding to specific receptors on their surfaces, which activates internal signaling pathways that induce cholesterol metabolism genes. These reactions may cause formation of foam cell production [102]. When TMAO was administered to mice peritoneal macrophages, researchers observed that it inhibited the RCT and reduced the amounts of hepatic BA production and BA transporters [103].

#### TMAO-induced vascular dysfunction

TMAO increases the expression of macrophage scavenger receptor cluster of differentiation 36 (CD36) and scavenger receptor class A type 1 (SR-A1), which causes foam cell production [104, 105]. It also promotes monocyte-to-macrophage differentiation and facilitates the uptake of oxidized LDL (oxLDL), contributing to atherosclerotic progression [106]. Additionally, it enhances the macrophage mobility, which aids in the development of AS’ advanced stage [107]. TMAO upregulates high mobility group box 1 (HMGB1), which in turn activates toll-like receptor 4 (TLR4), leading to downstream activation of ERK1/2 and NF-κB signaling pathways [108]. The TLR-4-NF-κB is implicated in many diseases and pathological states [109–112]. Notably, the molecular events triggered by TMAO disrupt endothelial integrity by reducing the expression of tight junction proteins Zonula Occludens-2 (ZO-2) and Occludin (OCLN), while decreasing phosphorylated endothelial nitric oxide synthase (p-eNOS), thus increasing endothelial permeability and allowing LDL to enter and oxidize to ox-LDL [113, 114].

Furthermore, TMAO promotes a pro-inflammatory state. Through the production of inflammatory cytokines, increased plasma TMAO levels increase the risk of inflammation and cardio-metabolic problems [115]. Mechanistic studies indicate that TMAO can stimulate hepatocytes to release exosomes that are taken up by vascular endothelial cells, inducing expression of inflammatory cytokines (including IL-6, MCP-1, and TNF-α), activating NF-κB signaling, increasing endothelial cell apoptosis, and impairing endothelium-dependent vasodilation [116]. Through the modulation of cholesterol and BA metabolism, activation of inflammatory pathways, and reduction of endothelial function, TMAO therefore links diet, gut microbial metabolism, and elevated cardiovascular risk. Preventing TMAO production or its downstream effects could be an effective strategy to slow the progression of CVD (Fig. 2).

### Specific microbial pathways

The initial step in microbial cholesterol metabolism involves the oxidation of cholesterol to cholestenone (4-cholesten-3-one), a reaction catalyzed by the enzyme cholesterol oxidase. This enzyme simultaneously reduces molecular oxygen to hydrogen peroxide (H₂O₂) during the oxidation process [117]. This enzyme, which may be influenced by phospholipid composition and surface pressure, is essential for the clinical assessment of serum cholesterol in order to diagnose lipid disorders [118, 119]. A wide range of bacteria, including *Streptomyces sp.*,* Bacillus pumilus*,* and Serratia marcescens*, are known to generate cholesterol oxidases [120–122]. In addition, *Mycobacterium species and Nocardia rhodochrous* express cholesterol oxidase as an intrinsic, membrane-bound enzyme localized on the exterior of the cell membrane [123, 124]. Whereas extracellular cholesterol oxidase is secreted into the broth medium by other genera such as *Pseudomonas*,* Arthrobacter*,* Rhodococcus equi*,* Brevibacterium sterolicum*, and *S. violascens* [125]. Notably, *Rhodococcus* sp. GK-1, a soil-isolated strain, has been shown to produce both extracellular and intracellular forms of the enzyme [126].

It is hypothesized that while non-pathogenic bacteria utilize cholesterol oxidase to degrade cholesterol as a nutrient source, pathogenic species exploit the enzyme to alter host lipid membranes and facilitate infection of macrophages [127]. Cholestenone and coprostanone are the intermediate products of colonic bacteria’s metabolism of cholesterol, whereas coprostanol is the end product. Although only a limited number of bacterial species, such as *Eubacterium coprostanoligenes* ATCC 51,222 and *Bacteroides* sp. strain D8, are known to efficiently convert cholesterol to coprostanol, the specific enzymes responsible for this biotransformation have yet to be fully elucidated [128, 129].

## Gut microbiota and host gene expression

The gut microbiota is crucially involved in regulating gene expression in the host by various pathways, including epigenetic modifications, transcriptional regulation, and post-transcriptional mechanisms. Beyond the confines of the gut, they influence metabolic intestinal homeostasis, immune responses, cardiovascular functions, and neurobiology [3, 4, 130]. Gut microbiota can influence the physiological as well as pathological processes of the host by modulating various signaling pathways, nuclear receptors, and gene expression programs. The intestinal epithelium, in conjunction with the mucus layer, constitutes the first physical barrier against gut microbes and interface for host–microbe signaling [131].

Epigenetic changes, including DNA methylation, histone modification, chromatin accessibility, and non-coding RNAs, are among the most significant ways that the gut microbiota may control gene expression in the host [132].

These changes affect chromatin accessibility and the recruitment of transcriptional machinery, which regulates gene activation or suppression. DNA methylation is an epigenetic regulatory mechanism in animals that affects gene expression by modulating the accessibility of transcription factors, histone modifiers, and transcriptional machinery to chromatin. This alteration is extensively controlled, including several components that add and remove methyl groups. DNA methyltransferases (DNMTs) facilitate the addition of a methyl group from the donor S-adenosylmethionine (SAM) to the carbon-5 position of cytosine (5mC), whereas the ten-eleven translocation enzyme (TET) dioxygenase family can actively reverse this modification by oxidizing 5mC to 5-hydroxymethylcytosine (5hmC). The production dynamics of 5mC and 5hmC are crucial for several biological activities and may be modulated by different small compounds [133, 134]. DNA methylation is the most important epigenetic mechanism and significantly affects the pathogenesis of AS [135]. A deficit in folic acid may enhance methylation, elevate homocysteine levels, impair vascular endothelial cells, and facilitate AS [136]. Folate and other B vitamins (B2, B12) that provide methyl groups for DNA or histone methylation are produced by the gut microbiota.

Exposure to commensal microbiota induced TET2/3-dependent localized DNA methylation alterations at intestinal epithelial regulatory sites [137]. Many commensal microorganisms, such as the probiotic *Bifidobacterium* and *Lactobacillus* species, generate folate. These bacteria engage in one-carbon metabolism to make S-adenosylmethionine (SAM), which is the main substrate for DNA and histone methylation [138, 139]. At the end of the treatment with *Bifidobacteria adolescentis MB 227*,* B. adolescentis MB 239*, and *B. pseudocatenulatum MB 116*, the level of serum folate in folate-deficient rats receiving both the probiotic and synbiotic diet was markedly elevated compared to the control group [140]. Furthermore, studies have demonstrated the ability of certain strains of *Lactiplantibacillus plantarum* and *Latilactobacillus sakei* to synthesize folate [141]. Data from 256 common human gut bacteria indicate that the folate production pathway was found in almost all *Bacteroidetes* genomes and the majority of *Fusobacteriota* and *Proteobacteria* genomes, but the *Firmicutes* and *Actinobacteria* families lacked these genes [142]. This study hypothesized that 37% of the daily required folate intake for individuals who are not pregnant or breastfeeding may be produced by the human microbiome.

The core nucleosome, which is surrounded by DNA, is composed of histones (H2A, H2B, H3, and H4). Histones can undergo a variety of covalent modifications, such as acetylation, methylation, phosphorylation, and ubiquitination, mainly on the N-terminal histone tails. These modifications are now recognized as essential regulatory characteristics of chromatin structure and function, which alter cell fate and tissue development [143]. Cardiovascular disorders may arise from imbalances in histone modifications. For example, the depletion of methylation and acetylation of histone H3 and H4 residues serves as a biomarker for AS [144].

SCFAs also affect the epigenome by modifying histones and DNA methylation. They can also influence the activity of ten-eleven translocation methylcytosine dioxygenase (TET) enzymes, which are essential for DNA demethylation and, if targeted at gene promoters, increase transcription [145]. Moreover, SCFAs inhibit host histone deacetylases (HDACs), which normally remove acetyl groups from histone lysines, resulting in chromatin condensation and transcriptional silencing [146].

When butyrate was administered directly to intestinal epithelial stem cells, which are located at the base of the crypts and are anatomically shielded from exposure to SCFAs, it inhibited HDAC activity and increased H3K9 and K27 acetylation, which was accompanied by decreased proliferation and a diminished ability to respond to injury [147, 148]. HDAC inhibition is associated with the upregulation of genes involved in immune regulation, vascular stability, and CVD onset [149]. In inflammatory settings, propionate, butyrate, and valerate may restore ApoA-I transcription via PPARα transactivation-mediated NF-κB suppression, therefore preventing development of AS [150–152].

## Gut microbiota contributions to AS and CVD

Recent evidence indicates that gut microbial ecology is closely associated with the onset and progression of CVD. Additionally, imbalances in microbe–host interactions can disrupt homeostasis, contributing to CVD development (Table 1). Moreover, the gut microbiota generates bioactive metabolites such as TMAO, SCFAs, and BAs, which are implicated in influencing host health via multiple pathways [153, 154].

**Table 1:** Key microbiota associations identified in cardiovascular diseases (CVD)

Author	Date	Study population	Disease	Analysis approach	Samples	Associated bacteria	Ref
Jie et al.	*2017*	*405*	ACVD	metagenome-wide association	Stool	*Enterobacteriaceae and Streptococcus* spp	[207]
Liu et al.	2019	201	CAD	16 S rRNA	Stool and serum metabolomics	*Koseburia*, *Klebsiella*, *Clostridium IV*, and *Ruminococcaceae*	[287]
Toya et al.	2020	266	CAD	16 S rRNA	Stool	*R. gnavus*	[288]
Verhaar et al.	*2020*	4672	HTN	16 S	Stool	*Roseburia* spp., *Clostridium* spp., *Romboutsia* spp.and *Ruminococcaceae* spp.	[289]
Wang et al.	*2021*	52	Chronic HF	16 S rRNA	Blood and Stool	*Escherichia Shigella*,* Haemophilus and Klebsiella*	[290]
Sun et al.	*2021*	59	Chronic HF	16 S rRNA	Stool	*Enterococcus and Enterococcaceae*	[291]
Zhang et al.	*2022*	80	Chronic HF	16 S rRNA	Stool and plasma	*Escherichia*,* Bifidobacterium*,* Klebsiella and Lactobacillus*	[292]
Sayols et al.	*2023*	*8973*	Coronary atherosclerosis	shotgun metagenomics sequencing	Stool	*Streptococcus anginosus* and *Streptococcus oralis*	[293]
Dong et al.	*2023*	*35*	coronary atherosclerosis	16 S rDNA	Stool	*Megamonas*,* Streptococcus*,* Veillonella and Ruminococcus torques*	[294]
Sato et al.	*2024*	54	Internal Carotid Artery Stenosis	16 S rRNA	carotid plaques and oral	*Streptococcus*,* Actinomyces*, and *Veillonella*	[295]

### Heart failure (HF)

HF, a clinical syndrome arising from cardiac dysfunction characterized by a diminished capacity of the heart to pump or fill with blood, often represents a late stage of CVD. At this stage the heart fails to satisfy the metabolic demands of the body, leading to symptoms such as dizziness, fatigue, dyspnea, and fluid retention [155, 156]. Research indicates that a number of processes that cause anatomical and functional abnormalities in the gastrointestinal tract, such as splanchnic congestion and modifications to the host’s immunological defense system, are related to the bacterial translocation in HF [157]. Increased bacterial translocation might worsen the course and prognosis of HF in people with the condition due to intestinal mucosal edema brought on by reduced cardiac output and severe venous blood congestion [158, 159]. Pathogen overgrowth increases the likelihood of gastrointestinal infections in HF patients, resulting in a worse in-hospital prognosis. Among HF patients hospitalized with infections, including urinary tract infection, pneumonia, or sepsis, those with concurrent *Clostridioides difficile* infection (CDI) had higher in-hospital mortality than those without CDI [160].

Research indicates that individuals with chronic HF may exhibit an overgrowth of pathogenic gut microbiota and heightened intestinal permeability [161]. Therefore, growing intestinal permeability and underlying systemic inflammation in HF are correlated with rising fecal bacterial counts [162]. There is evidence that people with HF have higher amounts of pro-inflammatory cytokines in their blood, including TNF, IL-1, and IL-6, which increases intestinal permeability [163]. Elevated plasma lipopolysaccharide (LPS) during edematous episodes has been implicated as a trigger for cytokine production and immune activation [164]. HF may result in intestinal barrier dysfunction and congestion of the splanchnic circulation, which would raise the number of bacterial products in the system’s bloodstream and exacerbate the inflammatory condition [165]. An increase in dangerous bacteria, such as *Campylobacter*,* Salmonella*,* Shigella*,* Yersinia enterocolitica*,* and Candida* species, and a reduction in beneficial butyrate-producing bacteria have been observed in chronic HF [166]. Also, a notable reduction in the families *Coriobacteriaceae*, *Erysipelotrichaceae*, and *Ruminococcaceae*, as well as a decrease in gut microbial diversity, was identified [167].

Gut microbiota metabolites, including SCFAs and TMAO, are also linked to HF. Research revealed that in individuals with HF, increased TMAO may be a negative prognostic factor [168–170]. Additionally, in HF with reduced ejection fraction (HFrEF), higher levels of TMAO were predictive of morbidity and death [171]. Thereby lowering the circulating TMAO level by intestinal microbiota or pertinent enzyme intervention for improving the prognosis of HF patients has emerged as a significant area of study [172].

Research demonstrated that SCFAs and BAs are also associated with HF. In a mouse model of transverse aortic constriction, the decrease of SCFAs and an increase in primary BAs were also shown to correlate with the development and severity of cardiac conditions [173]. Patients with HF appear to have higher levels of secondary BAs. Additionally, butyrate-producing bacteria, especially those belonging to the *Lachnospiraceae* and *Ruminococcaceae* families, have been shown to be reduced in HF patients [174]. Overall, the interplay between gut microbiota composition, microbial metabolites, and HF pathophysiology highlights strong associations between gut and cardiovascular health. Dysbiosis has been associated with systemic inflammation, increased intestinal permeability, and metabolic dysregulation, which may contribute to HF progression. Thus, changes in SCFA and BA metabolism suggest a role for the gut in modulating cardiac function. A holistic knowledge of the interactions will be important to identify biomarkers and risk factors associated with HF development and progression.

### HTN

HTN is a progressive CVD resulting from both hereditary and environmental factors, causing alterations in cardiac and vascular function and structure. In addition to genetic and environmental factors, the gut microbiota has been implicated in the pathophysiology of HTN [175]. In comparison to healthy persons, HTN patients exhibited a reduction in the quantity and variety of gut microorganisms, while the species *Prevotella* was markedly elevated [176]. Yang et al. found that hypertensive rat models exhibited gut dysbiosis, including reduced diversity and richness [177]. *Prevotella (various ASVs)*,* Megasphaera*,* Butyricoccus*,* Prevotellaceae*,* Faecalibacterium*,* Lachnoclostridium*,* Howardella*,* and g-UCG04* taxa have a positive correlation with BP, *while Prevotella (other ASVs)*,* Alloprevotella*,* and Streptococcus* show a negative correlation with BP [178]. Also, by using 16 S amplicon sequencing on 129 fecal samples, Dan et al. discovered that 18 genera, including *Alistipes*,* Bacteroides*,* Barnesiella*,* Butyricimonas*,* Christensenella*,* Clostridium sensu stricto*,* Cosenzaea*,* Desulfovibrio*,* Dialister*,* Eisenbergiella*,* Faecalitalea*,* Megasphaera*,* Microvirgula*,* Mitsuokella*,* Parabacteroides*,* Proteiniborus*,* Terrisporobacter*, and *Acetobacteroides*, were noticeably more prevalent in the hypertensive *group* [179].

BAs are implicated in BP regulation through multiple physiological pathways. For example, intervening with taurine or tauro-cholic acid rescued BA conjugation and reduced BP in hypertensive rats [180]. BA receptors, including the membrane receptor G protein-coupled bile acid receptor 1 (GPBAR1) and the nuclear receptor FXR, are key regulators of endothelial function. FXR activation enhances nitric oxide (NO) synthesis while suppressing endothelin-1 (ET-1) release, a potent vasoconstrictor implicated in BP regulation [181]. Additionally, TGR5 activation promotes internalization of ET-1 receptors in hepatic stellate cells (HSCs), thereby attenuating ET-1 responsiveness and reducing its expression and secretion from liver sinusoidal endothelial cells (LSECs) [182]. BAs also contribute to BP regulation by influencing renal water and electrolyte homeostasis [183]. FXR and TGR5 regulate aquaporin function in renal tubules, thereby modulating urine concentration and systemic fluid balance [184, 185]. Notably, reduced TGR5 activation has been associated with the development of HTN in stroke-prone, spontaneously hypertensive individuals. In the same study, supplementation with CA led to a mean reduction of 23 mmHg in BP over a six-week period, indicating there is therapeutic potential for BA modulation [186]. Moreover, BAs exert metabolic effects that indirectly influence HTN. They enhance insulin secretion, improve insulin sensitivity, and mitigate insulin resistance, key factors in the pathophysiology of HTN [187]. A positive correlation between elevated bile salt levels and HTN has been observed in diabetic patients, further supporting the interplay between BA metabolism and cardiovascular risk [188].

The absence of SCFAs-producing bacteria has been connected to the HTN [189]. One of the important functions of SCFAs is BP control, which is mostly accomplished by GPR41 and olfactory receptor 78 (Olfr78) [190]. Olfr78 may contribute to the development of HTN by the sympathetic nervous system’s activation and the rise in carotid body activity [191]. In addition, mice deficient in the *Gpr41* gene have been shown to have high BP [192]. HTN may also be associated with the intestinal metabolite TMAO. It was shown that an increase in TMAO levels of 5 and 10 µmol/L was associated with a 9% and 20% increase in the risk of HTN, respectively [106]. In Ang II-induced hypertensive mice, increasing levels of TMAO increased systolic BP and promoted vasoconstriction. Nevertheless, minimal quantities of TMAO do not influence BP [193]. Spontaneously hypertensive rats (SHR) were allocated to either the water group or the groups receiving water supplemented with 333 mg/L or 1 g/L TMAO. After 22 weeks, no significant change in diastolic BP was found; however, a tendency towards reduced systolic BP was seen with increasing dosages of TMAO in the SHR group [194].

Through the production of superoxide-stimulated oxidative stress and advanced glycation end-products, TMAO may cause aorta stiffening and raise systolic BP. This may happen as aging increases intrinsic wall stiffness [195]. Additionally, TMAO has also been associated with vascular endothelial dysfunction by promoting inflammation via nuclear factor-κB and mitogen-activated protein kinase signaling, which may lead to HTN [196]. Nevertheless, it is unclear what precise chemical mechanism TMAO uses to affect BP, and further research is necessary. Modulation of BP by microbiota-derived metabolites represents a potential therapeutic avenue under investigation for HTN management.

### AS

AS, the predominant cause of CVD, is a chronic, systemic, and inflammatory condition that mostly affects big and medium-sized arteries [197]. AS is characterized by thick arterial walls and persistent inflammation brought on by lipoprotein-rich plaques that develop on the vascular endothelium, restricting the arterial lumen and limiting blood flow [198, 199]. One risk factor for AS is a compromised intestinal barrier as a result of dysbiosis in the gut microbiota [200]. Determining the extent to which gut microbiota contribute to plaque development is an active area of investigation.

Detection of bacterial DNA in atherosclerotic plaques suggests possible involvement of microbes in CVD, although the directionality and mechanism remain uncertain [201]. Bacterial DNA in plaques may stimulate macrophages and activate the innate immune response via Toll-like receptors 2 (TLR2) and 4 (TLR4), perhaps correlating with plaque stability [202].

Some findings suggest the gut microbiota may be significantly associated with AS. An analysis using shotgun DNA sequencing focused on the gut metagenome that indicated significant changes in the diversity of gut microbial populations between patients with symptomatic AS and those assumed to be healthy controls [203]. In general, a high ratio of *Firmicutes*/*Bacteroidetes* may present as a marker of subclinical AS [204]. Individuals with AS showed considerably higher amounts of *Enterobacteriaceae*, *Ruminococcus gnavus*, and *Eggerthellalenta*, according to a comprehensive metagenome-wide association study conducted on a sample of 218 AS patients and 187 healthy controls [205]. Other studies also found that patients with coronary artery disease (CAD) and subclinical carotid AS had an enrichment of the phylum Escherichia [206]. A microbiome-wide association study (MWAS) involving stool samples from 218 atherosclerotic cardiovascular disease (ACVD) patients and 187 healthy controls revealed an increase in the prevalence of *Enterobacteriaceae* (including *Escherichia coli*,* Klebsiella spp.*, and *Enterobacter aerogenes*), bacteria typically found in the oral cavity (such as *Streptococcus spp.*,* Lactobacillus salivarius*,* Solobacterium moorei*, and *Atopobium parvulum*), and *Ruminococcus gnavus* [207].

TMAO may serve as an early indicator of AS. In other words, ASCVD patients have markedly elevated levels of TMAO [99]. As previously mentioned, dysbiosis has been associated with impaired intestinal barrier function, which could increase systemic exposure to TMA precursors that are then oxidized to TMAO in the liver [208]. TMAO has been linked to pro-inflammatory signaling, increases the production of foam cells, and encourages cholesterol collecting in macrophages, which all lead to the development of plaque in the arteries [209]. A community-based cohort of 4131 older US adults for incident ASCVD and 1449 for recurrent ASCVD found that higher plasma levels of TMAO were associated with an increased risk of ASCVD [210]. Additionally, research that investigated the relationship between TMAO and neo-AS found that in patients with very late stent thrombosis, plasma level of TMAO was substantially linked to both new AS and plaque rupture [211].

SCFAs are recognized for their ability to preserve intestinal barrier integrity, inhibit pathogen invasion, and reduce inflammation. ApoE−/− mice administered exogenous butyrate exhibited a greater than 50% reduction in aortic atherosclerotic plaques, characterized by decreased macrophage counts, higher collagen levels, and lowered inflammatory markers, indicating a possible protective function of SCFAs in AS [212]. SCFA-producing bacteria, such as *Roseburia intestinalis* and *Faecalibacterium prausnitzii*, were shown to be less prevalent in patients with ASCVD than in healthy controls [207]. In fact, SCFAs represent a potential therapeutic target for AS via modulation of lipid metabolism and inflammation, but clinical data are limited [213].

BA sequestrants have been reported to regulate blood cholesterol levels, thereby influencing the formation of atherosclerotic plaques. Beyond their lipid-lowering effects, other bile acid-related mechanisms also contribute to affecting the formation of atherosclerotic plaques [214]. For instance, synthetic FXR ligands have demonstrated anti-inflammatory potential. In rat smooth muscle cells, FXR activation inhibited the inflammatory response induced by interleukin-1β, suggesting that FXR agonists may possess anti-atherosclerotic properties [215]. Similarly, activation of the TGR5 in bovine aortic endothelial cells suppressed NF-κB activity while enhancing NO production. This, in turn, reduced monocyte adhesion, macrophage lipid accumulation, and intraplaque inflammation, thereby preventing atherosclerotic plaque buildup in the arteries [216]. Further supporting this protective role, oral administration of CDCA derivatives to apolipoprotein E-deficient mice led to a remarkable 95% reduction in aortic plaque formation. This was accompanied by a significant decrease in the expression of pro-inflammatory cytokines such as IL-6 and IL-1 within the aortic tissue [217]. Additionally, activation of FXR and TGR5 has been linked to both anti-inflammatory and lipid-lowering effects, contributing to atheroprotection in low-density lipoprotein receptor knockout mice [21].

## Targeted therapies

**Fig. 3:**
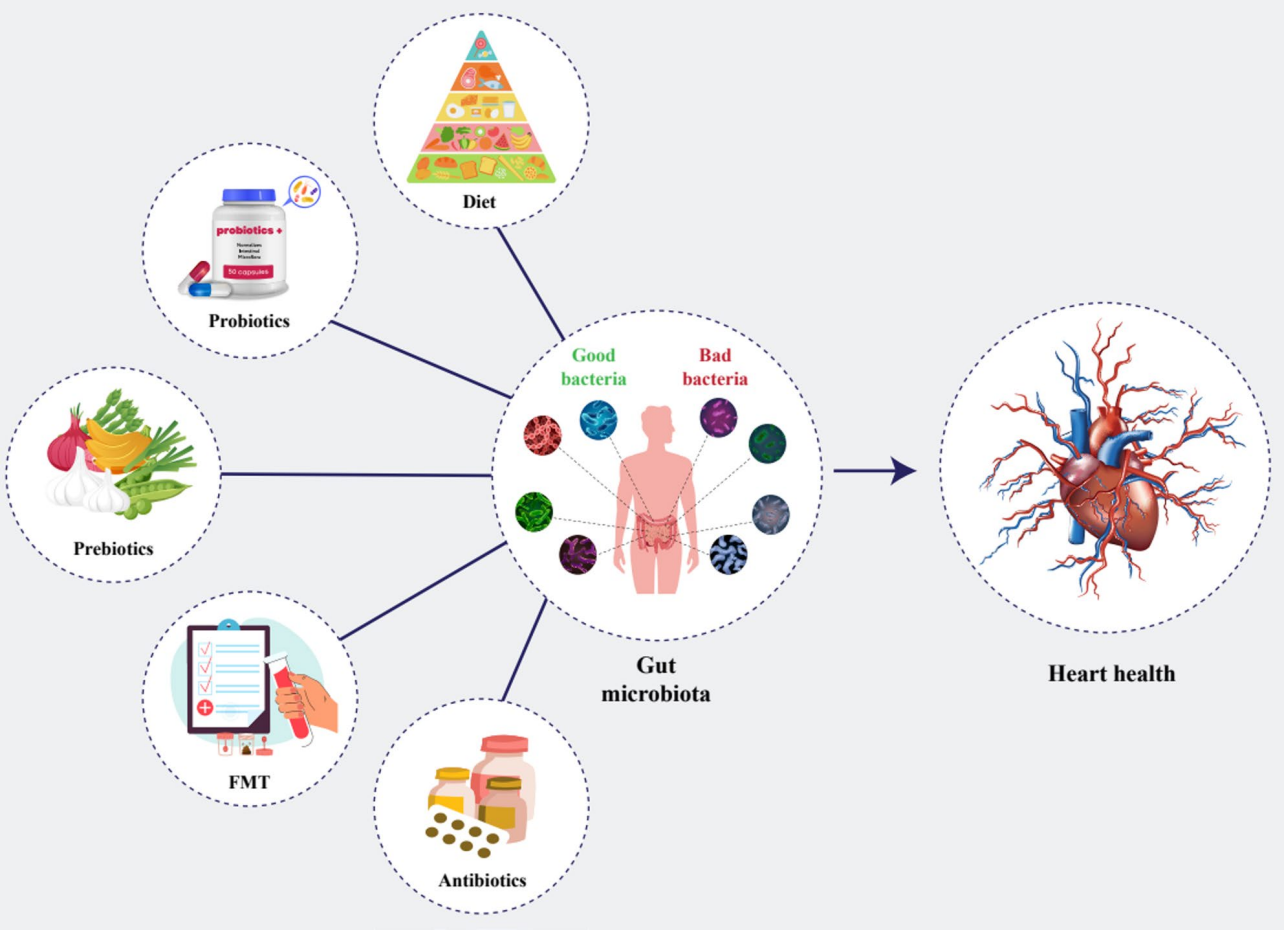
Modulation of gut microbiota and its impact on heart health. This schematic illustrates how diet, probiotics, prebiotics, fecal microbiota transplantation (FMT), and antibiotics shape the composition of the gut microbiota by influencing the balance between beneficial and harmful bacterial species. Alterations in this microbial community drive changes in metabolite production, immune signaling, and systemic inflammation. Such microbial-driven pathways converge on the cardiovascular system, modulating vascular function and cardiac performance

In recent years, many studies have shown that the composition and diversity of gut microbiota can be beneficially altered by diet, prebiotics, probiotics, and fecal transplantation to confer benefits to the host (Fig. 3).

### Dietary approaches

Dietary behaviors may affect the diversity of gut microbiota, which subsequently influences host health via the digestion and absorption of nutrients [218]. The gut bacterial population is influenced by a diet high in plant products, which promotes the development of fiber-fermenting species and increases the synthesis of phosphatidylcholine and SCFAs. On the other hand, a high-fat diet causes adverse alterations in the fecal metabolomic composition, systemic inflammation, and gut microbiota [120]. Intake of animal protein, amino acids, and saturated fats was linked to the *Bacteroides* enterotype, according to research that linked long-term dietary patterns with gut microbial enterotypes. The *Prevotella* enterotype, on the other hand, was rich in simple sugars and carbs but low in these categories [219, 220]. According to a systematic review of 19 studies, restricted diets raised *Bacteroides* and lowered triglycerides, whereas plant-rich diets increased *Faecalibacterium* and decreased total cholesterol. Diets high in polyphenols had an impact on *Alistipes* and *Ruminococcaceae* UCG 005 [221]. These findings suggest that diet composition associated in the establishment of gut microbial enterotypes and their metabolic health consequences.

The Mediterranean diet (MedDiet) has several advantages, including the reduction of blood cholesterol and inflammatory markers [222]. This diet is characterized by a high intake of fruits, vegetables, legumes, unrefined grains, and nuts; a moderate intake of fish, poultry, and dairy products (primarily cheese and yogurt); a low intake of red meat; the use of olive oil as the primary fat source; and regular but moderate consumption of wine. After three years of follow-up of 285 individuals who were randomly assigned to receive a low-fat diet (LFD), MedDiet supplemented with nuts, or MedDiet supplemented with extra-virgin olive oil, results indicate that inflammatory biomarkers such as TNF-α, IL-1β, IL-6, and hs-CRP were significantly reduced as a consequence of both MedDiet treatments. Also, the LFD group showed a tendency toward higher CXCR2 and CXCR3 expression, but no discernible alterations in gene expression were found [223]. By adherence to the MedDiet, increased *Faecalibacterium*, *Prevotella*, and *Bacteroides* were reported, along with improved glycemic control, reduced fat mass, and decreased inflammation [224]. Furthermore, among a well-delineated group of 60 healthy adults. Through 16 S rRNA sequencing, the researcher discovered that a stronger adherence to the MedDiet was associated with enhanced alpha diversity and a larger prevalence of *Paraprevotella* and *Bacteroides* [225]. Thus, adherence to the MedDiet improves aspects of inflammatory and metabolic health as well as consistently increases microbial diversity and abundance of beneficial taxa.

In a study with 153 subjects, researchers discovered that fecal SCFAs levels rose when participants had more fruits, vegetables, and legumes, which is in line with the MedDiet. Fermentation by a higher number of bacteria belonging to the *Firmicutes* and *Bacteroidetes* families is the cause of this impact [226]. As demonstrated in the Wang et al. research, following the MedDiet would significantly affect the gut microbiota in relation to the production of SCFA, secondary BA, and degradation of plant-derived polysaccharides. Additionally, the absence of *P. cpori* in the gut microbiota of a group of participants may be the cause of their protection against heart metabolic diseases [227]. This evidence suggests that SCFA production may be a mechanistic link between the MedDiet, gut microbiota and cardiometabolic protection.

Furthermore, promoting the MedDiet and controlling conventional cardiovascular risk factors may be a useful public health strategy to stop AS and lessen the prevalence of CVD [228, 229]. In comparison to an LFD, long-term use of a MedDiet high in extra virgin olive oil was linked to slower development of AS, as seen by lower carotid plaque height and intima-media thickness of the common carotid arteries [230]. The MedDiet markedly decreased postprandial glucose and insulin responses while enhancing insulin sensitivity compared to the Western diet (WD). These modifications were associated with elevated plasma butyric acid concentrations and changes in gut microbiota, including *Intestinimonas butyriciproducens* and *Akkermansia muciniphila* [231]. According to Aziz et al., WD rich in saturated fats and refined carbohydrates, causes dysbiosis by promoting the proliferation of pro-inflammatory microorganisms and decreasing microbes that produce SCFAs [232]. Additionally, increased arterial stiffness, endothelial dysfunction, intestinal dysbiosis, and higher inflammatory markers were seen in male C57BL/6J mice that were given a WD for 7 months [233]. These results indicate the MedDiet as a protective dietary model against the Western diet, with many of the benefits occurring through changes in the gut microbiota. Diet-based interventions, especially the MedDiet, confer beneficial cardiometabolic effects by increasing gut microbial diversity, increasing SCFA production, and mitigating the deleterious effects of a Western dietary pattern.

### Probiotics

Probiotics are defined as live strains of carefully chosen microbes that, when given in sufficient quantities, enhance the host’s health [24, 234]. A large body of evidence during the recent decades have unveiled extensive applications of probiotics as therapeutic agents, therapeutic targets, and drug delivery vehicles [235–239]. By competing with pathogens, probiotics may influence the dynamics of the current microbial population. Probiotics need to meet the following requirements: [1] be living microorganisms; [2] be able to be kept alive and stable before use; [3] be resistant to the digestive process; [4] be demonstrated to be advantageous to the host by science; and [5] be a part of the original intestinal microbiota or be safe and dependable. In actuality, a lot of goods on the market do not meet these fundamental requirements [240]. Therefore, standardized criteria are essential to ensure the therapeutic reliability of probiotic preparations. Probiotics may be an effective additional therapy for obesity and lipid metabolism management by regulating the gut microbiome. Table 2 summarizes preclinical studies in rodent and swine models demonstrating how specific probiotic strains, dosages, and treatment durations modulate BP, lipid metabolism, oxidative stress pathways, and inflammatory markers in CVD contexts. Through the establishment of a healthy gut microbiota or the supplementation of its metabolites, probiotics are useful in enhancing heart health [241]. These supplements prevent plaque formation and have an anti-atherosclerotic impact by increasing cholesterol utilization and displaying anti-inflammatory qualities [242]. It has been proposed that *Lactobacillus* and other probiotics help the body metabolize cholesterol while it is growing, which lowers cholesterol levels [243]. Hence, probiotic interventions appear to target multiple atherogenic pathways simultaneously, including lipid regulation and inflammation.

**Table 2:** Probiotics administration and cardiovascular outcomes in preclinical models

Author	Date	Background	Bacteria	CFU	Duration	Outcome	Ref
Robles-Vera et al.	2020	Spontaneously hypertensive rats	*B. bifidum* and *l. fermentum cect5716*	1 × 10^9^	13 weeks	prevent the BP increase ↓NADPH oxidase-driven reactive oxygen species production ↑Treg infiltration	[296]
Hassan et al.	2020	atherosclerosis-prone ApoE^−/−^ mice	*L. plantarum ATCC 14,917*	2 × 10^9^	12 weeks	inhibited atherosclerotic lesion formation ↓e oxLDL, MDA, TNF-α and IL-1β ↑SOD attenuated IκBα protein degradation inhibited the translocation NF-κB.	[297]
Jiang et al.	2020	high-fat high-cholesterol fed ApoE^−/−^ mice	*L. mucosae A1*	1 × 10^9^	13 weeks	attenuated the severe lipid accumulation in serum, liver and aortic sinus ↓serum lipopolysaccharide-binding protein	[298]
Aboulgheit et al.	2021	Yorkshire swine ( An ameroid constrictor was placed around the left coronary circumflex artery)	*L. plantarum*	2 × 10^7^	14 weeks	activate of Nrf2 mediated pathways Improve vascular function and diastolic dysfunction, ↑eNOS	[299]
O’Morain et al.	2021	High-fat-fed LDLR -/- C57BL/6J mice	*L. plantarum CUL66*	5 × 10^8^	12 weeks	↑ HDL and ↓LDL/VLDL levels ↓plaque burden indicative of dampened inflammation, ↑smooth muscle cell content ↓ macrophages and T-cells number in bone marrow	[300]
Wang et al.	2022	C57BL/6 mice fed a Paigen atherogenic diet	*L. plantarum*,* L. reuteri*,* L. casei*,* B. breve*,* and B. adolescentis*	1 × 10^9^	16 weeks	↓TC, LDL–c, AST, ALT and TMAO ↑the faecal SCFAs Alleviate of hypercholesterolaemia.	[301]
Zhai et al.	2022	ApoE−/− mice fed a high-fat diet	*L. rhamnosus* GG	1 × 10^7^ once a week	12 weeks	↓endothelial injury improving ketone body synthesis	[302]
Wang et al.	2023	spontaneous hypertensive rat	*C. butyricum pMTL007- GLP-1*	1 × 10^9^ every 2 days	6 weeks	↓BP improved cardiac marker ACE2, AT2R, AT1R, ANP, BNP, β-MHC, α-SMA activatie AMPK/mTOR/p70S6K/4EBP1 signalling pathway improved the dysbiosis	[303]
Luo et al.	2023	Spontaneously Hypertensive Rats	*C. butyricum CGMCC 1.5205*	1 × 10^8^	6 weeks	↑Total SCFA especially butyrate prevented the adverse effects of SHR on intestinal flora, vascular, and BP	[304]
Yang et al.	2024	CHD	*L. plantarum P470*	1 × 10^9^	4 weeks	Enhanced hydroxyl radical scavenging and exhibited anti-inflammatory effects on macrophages regulated the biosynthesis of unsaturated fatty acids and linoleic acid metabolism improve the fecal physiological status	[305]
Zhu et al.	2024	high-fat diet ApoE^−/−^ mice	*E. faecium NCIMB11508*	2 × 10^9^	12 weeks	Inhibit the formation of atherosclerotic lesions. ↓the inflammatory factor ↓LPS-induced damage ↑SCFAs	[306]
Yang et al.	2024	fed a high-fat diet ApoE^−/−^ mice	*F. prausnitzii ATCC 27,766*	2.5 × 10^9^ CFU/100 µL	12 weeks	↓ inflammation Anti-atherosclerotic effect ↓ LPS in plasma	[307]
Hassan et al.	2024	fed a high-fat diet ApoE^−/−^ mice	*L. plantarum ATCC 14,917*	2 × 10^9^	12 weeks	↓inflammatory markers (ICAM-1, CD-60 MCP-1, F4/80, ICAM-1, and VCAM-1) in the thoracic aorta, (Ccr7, cd11c, cd4, cd80, IL-1β, TNF-α) in the colon ↑ ROS-scavenging enzymes activity (SOD-1, SOD-2) ↓ athero plaque size ↓ LPS	[308]
Liu et al.	2025	fed a high-fat diet ApoE^−/−^ mice	*L. rhamnosus GG*	2 × 10^9^	15 weeks	Inhibit of atherosclerosis progression ↓oxidative stress and inflammation. ↑ Nrf2/HO-1 ↓aortic macrophages ↑ Tregs	[309]
Liu et al.	2025	high-fat diet Mice	*E. coli Nissle 1917*	1 × 10^9^	12 weeks	alleviated atherosclerotic plaque and lipid droplet production. ↓TC, TG, HDL and LDL. ↓ pyroptosis-related markers (cleaved Caspase-1, GSDMD-N, NLRP3, IL-18, IL-1β)”	[310]

Furthermore, a high-fat diet may lower plasma cholesterol levels by consuming the Lab4 probiotic consortium, which includes *Bifidobacterium bifidum*, *Bifidobacterium animalis subsp*. *lactis*, and two strains of *Lactobacillus acidophilus*, together with *Lactobacillus plantarum* CUL66 (Lab4P) [244]. In rats suffering from an acute myocardial infarction brought on by a permanent coronary artery blockage, oral therapy of the probiotic *Lactobacillus rhamnosus* GR-1 improved cardiac remodeling and pump failure [245]. Additionally, atherosclerotic plaque development was decreased when ApoE-deficient mice were treated with *B. vulgatus* and *B. dorei*, two species that may be less abundant in CAD patients [246]. A double-blind clinical experiment with 44 CAD patients showed significant weight improvement and a strong anti-inflammatory impact after using *L. rhamnosus* supplements for 12 weeks while following a calorie restriction [247]. Similarly, it has been found that *Lactobacillus paracasei* shows promise for antihyperglycemic, anti-inflammatory, and lipid-lowering characteristics in patients with AS when given to BALB/c mice on a high-fat diet [248]. Patients with type 2 diabetes who took probiotic supplements had reduced BP, according to a meta-analysis of data from 11 randomized controlled trials [249]. More research discovered that the capacity of probiotics to produce bioactive peptides, such as angiotensin-converting enzyme inhibitory peptides, may account for their capacity to lower BP [250]. These findings support the translation of probiotic therapy from preclinical to clinical contexts, with consistent benefits across cardiovascular risk factors.

Additionally, an umbrella meta-analysis of randomized controlled studies revealed that taking probiotic supplements dramatically lowered systolic BP and diastolic BP levels [251]. Furthermore, probiotics also control gastrointestinal chemicals linked to CHD. improved lipid metabolism, reduced TMAO-induced AS, and controlled TMA-TMAO in serum and cecum by using *Lactis F1-3-2* intervention on the mice with a high-choline diet [252]. A pilot study with 21 participants suffering from stable CAD showed that a 6-week daily administration of *L. plantarum 299v* significantly affects CVD by inducing changes in gut microbiome-derived circulating metabolites. Probiotic supplementation may improve endothelium-dependent vasodilation of the brachial artery by augmenting nitric oxide bioavailability and reducing systemic inflammation. These effects seem to be independent of traditional cardiovascular risk factors and not associated with TMAO serum levels [253]. Therefore, the application of probiotics to modify the composition of the gut microbiota is a potential therapeutic target for CVD. It is important to note that while probiotics show promise, their effectiveness is limited. Probiotic effects are often highly strain-specific, meaning benefits observed with one strain may not apply to others, even within the same species [254]. Some studies demonstrated that different strains of the same species can have distinct impacts on immune modulation, gut microbiota composition, and clinical outcomes, which points to the importance of careful strain selection and characterization in both research and clinical use [255, 256]. This aspect makes it difficult to generalize findings or recommend specific strains for particular conditions. In addition, optimal dosing for probiotics remains unclear. While higher doses are sometimes associated with greater efficacy, the relationship is not always linear, and effective doses can vary by strain, disease, and host factors [257]. Additionally, the duration of administration can influence outcomes, with some benefits only emerging after prolonged use [258]. Thus, more well-designed, standardized, and personalized studies are needed to optimize probiotic interventions for diverse populations. Table 3 presents clinical trials of probiotic supplementation in patients with coronary heart disease, metabolic syndrome, obesity, and related disorders, highlighting effects on lipid profiles, glycemic control, inflammatory biomarkers, and surrogate cardiovascular outcomes.

**Table 3:** Outcomes of probiotics therapy in patients with cardiovascular and metabolic disorders

Author	Date	Study population	Background	Bacteria	CFU	Duration	Outcome	Ref
Raygan et al.	2019	54	Diabetic with CHD	*L. acidophilus*,* L. reuteri*,* L. fermentum*,* B. bifidum* (co-supplementation with 200 µg/day selenium)	8 × 10^9^	12 weeks	↓ Beck Depression and Anxiety ↓FPG, serum insulin levels, insulin resistance, TG, TC, VLDL, hs-CRP ↑NO, TAC, GSH ↓ Atherosclerosis formation	[311]
Depommier et al.	2019	40	Overweight/obese insulin-resistant	*A. muciniphila*	10^10^	12 weeks	↑insulin sensitivity, ↓insulinemia and plasma TC ↓body weight, and fat mass and hip circumference ↓the levels of relevant blood markers of liver dysfunction and inflammation ↓ Atherosclerosis formation	[312]
Moludi et al.	2021	44	MI	*L. Rhamnosus GG* (co-supplementation with insulin)	1.6 × 10^9^	12 weeks	↓TGF-β and TMAO levels Improvements in echocardiographic indices.	[313]
Chiu et al.	2021	40	mild hypercholesterolemic	*L. acidophilus La5*,* L*,* casei TMC and B. lactis Bb12*	2 × 10^6^	12 weeks	improved the fecal weight, fecal movement by improving intestinal microbiota and lag time of low-density lipoprotein (LDL) oxidation. ↓TC and LDL-c.	[314]
Koopen et al.	2022	12	MetS	*A. soehngenii L2-7*	-	24 h	↑ GLP-1 ↑secondary bile acids diminished peripheral glucose	[315]
Sun et al.	2022	60	CAD	*B. lactis Probio-M8*	3 × 10^10^	24 weeks	↓IL-6 and LDLc ↓TMAO and proatherogenic amino acids	[316]
Lee et al.	2024	99	obese	*B. lactis IDCC 4301*	5 × 10^9^	12 weeks	↓BMI total fat, leg fat, trunk fat and TG	[317]
Luangphiphat et al.	2025	58	MetS	*L. paracasei MSMC39-1 and B. animalis TA-1*	1 × 10^9^	12 weeks	↓LDL-c, body weight, body mass index, WC, SBP, and TC	[318]

### Prebiotics

Prebiotics are substances that undergo selective fermentation and produce certain alterations in the gastrointestinal microbiota’s composition and activity, which benefits the host’s health [259]. Prebiotics are chosen based on a number of factors, including [1] resistance to digestion in the upper parts of the digestive tract; [2] selective fermentation by the colon’s potentially advantageous microbiota; [3] positive impact on host health; [4] selective stimulation of probiotic growth; and [5] stability under different food/feed processing conditions [260]. Several researchers have shown that taking the right prebiotics may enhance health and safeguard the cardiovascular system [261]. For example, in patients with cardiovascular problems, prebiotics such as chitosan oligosaccharides, pectin polysaccharides, fructooligosaccharides (FOS), galactooligosaccharides, inulin, betaglucan, and minolest are often utilized. The proliferation of beneficial bacteria, the improvement of leaky gut by enforcing gut epithelial connections, and the stimulation of SCFAs production in gut bacteria are some of the several underlying processes that have been proposed [262]. These mechanisms illustrate how prebiotics can modulate gut microbiota composition to support cardiovascular health.

Clinical studies have further demonstrated the beneficial effects of prebiotics. Following the administration of inulin and a placebo, a randomized controlled experiment in human diabetes patients revealed a substantial decrease in proinflammatory cytokines and a promising improvement in lipid profiles [263]. When 19 g of inulin per day was administered to individuals with chronic renal disease for 6 months, the levels of serum insulin, fasting glucose, total cholesterol, triglycerides, CRP, and homocysteine decreased, but the levels of HDL cholesterol rose [264]. A systematic review was conducted on 13 studies, which included 513 adult participants. Prebiotic treatment decreased plasma total cholesterol and LDL-c levels in the overall study, whereas it lowered triglycerides and elevated HDL-c levels in diabetes trials [265]. Fructo-oligosaccharide and biotin supplementation enhance the variety of the microbiome and the capacity for bacterial synthesis of biotin and B vitamins in mice given a high-fat diet while preventing weight gain and glycemic reduction [266]. Despite these promising findings, a comprehensive examination of the microbiome in 14 obese men revealed that prebiotic treatment did not lead to significant weight reduction in obesity patients. This lack of effect may be attributed to the variability in each patient’s microbiota [267]. Therefore, prebiotic interventions are generally effective for metabolic and inflammatory parameters, although interindividual microbiota variability may influence outcomes.

Prebiotic interventions have also shown positive effects in HF and CAD. In an experimental HF model, rats had higher levels of endotoxin, lactobacilli indicators, and opportunistic microbes, whereas those treated with a prebiotic complex showed much lower levels. Markers of *propionibacteria*, *eubacteria*, and *bifidobacteria* showed reciprocal dynamics [268]. CAD patients who received chitosan oligosaccharides showed a substantial increase in the development of beneficial bacteria, including *Faecalibacterium*,* Lactobacilli*,* Escherichia*,* Alistipes*,* Phascolarctobacteria*,* and Lactococci* [269]. However, the response to prebiotics is highly dependent on the existing microbial strains in the host’s gut. Different individuals harbor distinct gut microbiota compositions, leading to variable responses to the same prebiotic. For example, the ability to utilize FOS or inulin depends on the abundance and types of *Bifidobacterium* and *Bacteroides* present, with some microbiota profiles showing greater benefit from prebiotic supplementation than others [270]. This means that the same prebiotic may not be equally effective across all individuals due to differences in microbial strain presence and abundance. In summary, prebiotics hold significant potential as a therapeutic strategy for CVD by modulating gut microbiota and promoting metabolic health. Further research is warranted to optimize their use and determine individualized treatment strategies for maximum efficacy.

### FMT

FMT is the procedure involving the direct transfer of healthy microbiota from a donor to the gut of a recipient with dysbiosis, aimed at restoring the normal composition and function of intestinal microbiota [271]. This intervention has demonstrated significant potential in the treatment of a number of cardiovascular and metabolic disorders (Table 4). FMT may improve metabolic indicators, lower systemic inflammation, and improve gut health by reestablishing the balance of the gut microbiota [272]. Emerging evidence suggests that FMT may also play a role in reducing myocardial inflammation. FMT may lower inflammation in cardiomyocytes and get rid of the elevated *Bacteroides/Firmicutes* ratio, which will reduce myocarditis [273]. *Firmicutes* and *Ruminococcaceae* were found to be more abundant in atherosclerotic mice after they received FMT from healthy mice. However, healthy mice that were fed FMT showed a rise in *Bacteroidetes* and a drop in *Firmicutes* and *Ruminococcaceae* [274]. Hence, FMT can restore a healthier microbial composition with potential cardioprotective effects.

**Table 4:** Preclinical and clinical fecal microbiota transplantation (FMT) interventions in CVD

Author	Date	Model	Delivery method	Type of study	Follow up	Outcome	Ref
Hu et al.	2019	autoimmune myocarditis mouse	Oral gavage	In vivo	3 weeks	↓IFN-γ expression (heart) & CD4⁺IFN-γ⁺ cells (spleen)	[319]
Kim et al.	2018	Obese or hypertensive mice	Oral gavage	In vivo	11 days and 2 weeks	↓inflammation in the colon improve glucose homeostasis ↓SBP	[273]
De groot et al.	2019	22 metabolic syndrome subjects	N/A	RCT	8 weeks	↓ insulin sensitivity ↑lithocholic, deoxycholic and (iso)lithocholic ↓ CCL2	[320]
Allegretti et al.	2020	22 obese subjects	FMT capsules	RCT	12 weeks	↓ taurocholic acid in stool Bile acid profiles become like	[321]
Kim et al.	2022	atherosclerosis-prone mouse	oral injection	In vivo	3 weeks	↓ formation of atherosclerotic lesions in the carotid arteries	[274]
Hatahet et al.	2023	obesity associated pre-HFpEF mouse	Oral gavage	In vivo	2 weeks	↑ systolic & diastolic function ↓inactive p-BCKDH in the heart	[322]
Lin et al.	2024	80 overweight and 106 normal weight subjects	Washed microbiota transplantation	RCT	N/A	↓ BMI ↓ ASCVD risk	[323]
Liang et al.	2024	56 subjects with hyperlipidemia and 68 subjects with normal lipids	Washed microbiota transplantation	RCT	1534 day	↓TC, TG, LDL-c and non-HDL-c ↓ ASCVD risk	[324]

FMT has also been linked to BP regulation. In an experimental study. After 8 weeks, germ-free mice who got FMT from a hypertensive patient showed higher systolic and diastolic BP than germ-free mice that received FMT from normotensive donors. They also formed a gut microbiota that was comparable to the donor’s [176]. Furthermore, in a pre-HFpEF animal model linked to obesity, oral FMT gavage improved both systolic and diastolic early dysfunction, indicating that butyrate is a key factor in this improvement [275]. Washed microbiota transplantation (WMT) is an enhanced technique of FMT that utilizes an automated purification system to cleanse and prepare the microbiota, with the objective of ensuring a safer and more accurate administration of beneficial gut bacteria to patients [276]. In metabolic syndrome patients, WMT therapy improved blood glucose, lipid profile, BP, and BMI and dramatically restored gut microbial equilibrium. WMT significantly reduced the risk of ASCVD in the high-risk metabolic syndrome patient group and significantly improved the risk of metabolic syndrome patients [277]. Additionally, prior studies have shown that WMT may help drop BP in hypertensive individuals, particularly in those who do not use antihypertensive medications [278]. Even while FMT has been indicated to have positive impacts on metabolic parameters, some studies have reported that it does not always result in clinically significant improvements in cardiometabolic disorders and fails to change intestinal microbiota [279, 280]. Given the positive outcomes observed in existing studies, microbiota transplantation holds great potential for future clinical applications. However, research on microbiota transplantation remains limited, and uncertainties persist regarding its variable efficacy and safety [276, 281]. More rigorous, standardized, and long-term studies are needed to optimize these interventions.

### Antibiotics

The use of antibiotics has the potential to reduce the presence of toxic bacteria, control the metabolites of the microbiota in the gut, and improve the prognosis of CVD [282]. Studies suggest that antibiotic therapy may positively influence cardiovascular risk factors. For instance, in a mouse model, ampicillin treatment led to a reduction in LDL and VLDL levels, which consequently resulted in fewer aortic atherosclerotic lesions [283]. Similarly, following triple antibiotic treatment, the increased systolic and diastolic BP in salt-induced hypertensive rats dramatically dropped [284].

Broad-spectrum antibiotics, such as vancomycin, rifampicin, and levofloxacin, have also demonstrated potential in treating resistant HTN. Animal studies in rats suggest that these antibiotics may modulate gut microbiota and initiate BP regulatory mechanisms. However, the precise mechanism by which antibiotics influence BP remains unclear and requires further investigation [285]. Despite these potential benefits, the use of broad-spectrum antibiotics raises concerns due to their adverse effects and the risk of bacterial resistance, making their role in reducing cardiovascular risk controversial [166]. Data from 26,000 CAD patients treated with quinolones and macrolides were examined in a Cochrane meta-analysis. According to the results of this meta-analysis, individuals with CAD did not experience a decrease in cardiovascular events while using antibiotics. On the other hand, individuals who got antibiotics had a much higher death rate, indicating that these drugs may have a negative effect on CAD [286]. While antibiotics may offer certain cardiometabolic benefits by modulating gut microbiota, their widespread use remains debatable due to potential risks. Thus, antibiotics are not currently recommended as a therapeutic strategy for CVD despite mechanistic potential observed in preclinical studies. Further research is needed to clarify their mechanisms, assess long-term safety, and determine their clinical utility in CVD management.

Taken together, the targeted therapies, dietary changes, probiotics, prebiotics, FMT/WMT, and antibiotics have demonstrated some degree of effectiveness in altering gut microbiota and improving cardiovascular health. Dietary changes, probiotics, and prebiotics have been shown to be the most effective, while FMT/WMT shows promising, but experimental potential, and antibiotic therapies are limited with regard to safety and the evidence base in clinical practice. These emphasize the gut microbiota as a critical modifiable target for innovative therapeutic strategies in CVD.

## Conclusion

In conclusion, the complex relationship between the gut microbiota and cholesterol metabolism represents a promising frontier in the understanding and treatment of CVD, particularly atherosclerotic diseases. As research continues to uncover the multifaceted roles of gut bacteria in regulating cholesterol levels and influencing cardiovascular health, studies suggest that targeting the gut microbiome could serve as an innovative therapeutic strategy. In this regard, future therapeutic strategies may utilize microbiota-modulating approaches to enhance cardiovascular health. These interventions can not only help lower cholesterol levels but also reduce inflammation and improve overall metabolic profiles, thereby reducing the risk of CVD.

## Data Availability

No datasets were generated or analysed during the current study.
